# Comparative effectiveness of adding weight control simultaneously or sequentially to smoking cessation quitlines: study protocol of a randomized controlled trial

**DOI:** 10.1186/s12889-016-3231-6

**Published:** 2016-07-22

**Authors:** Terry Bush, Jennifer Lovejoy, Harold Javitz, Brooke Magnusson, Alula Jimenez Torres, Stacey Mahuna, Cody Benedict, Ken Wassum, Bonnie Spring

**Affiliations:** Alere Wellbeing (now Optum), 999 3rd Ave, Seattle, WA 98104-1139 USA; Arivale, Inc. and University of Washington School of Public Health, 616 First Ave, Suite 700, Seattle, WA 98104 USA; SRI International, 333 Ravenswood Ave, Menlo Park, CA 94025-3493 USA; Bill and Melinda Gates Foundation, 440 5th Ave N, Seattle, WA 98109 USA; Center for Behavior and Health, Feinberg School of Medicine, Northwestern University, 680 N. Lakeshore Drive, Suite 1220, Chicago, IL 0611-8708 USA

**Keywords:** Smoking, Weight management, Combined treatment, Simultaneous, Sequential, Quitlines

## Abstract

**Background:**

Prevalence of multiple health risk behaviors is growing, and obesity and smoking are costly. Weight gain associated with quitting smoking is common and can interfere with quit success. Efficacy of adding weight management to tobacco cessation treatment has been tested with women in group sessions over an extended period of time, but has never been tested in real-world settings with men and women seeking help to quit. This paper describes the Best Quit study which tests the effectiveness of delivering tobacco and weight control interventions via existing quitline infrastructures.

**Methods:**

Eligible and consenting smokers (*n* = 2550) who call a telephone quitline will be randomized to one of three groups; the standard quitline or standard quitline plus a weight management program added either simultaneously or sequentially to the tobacco program. The study aims to test: 1) the effectiveness of the combined intervention on smoking cessation and weight, 2) the cost-effectiveness of the combined intervention on cessation and weight and 3) theoretically pre-specified mediators of treatment effects on cessation: reduced weight concerns, increased outcome expectancies about quitting and improved self-efficacy about quitting without weight gain.

Baseline, 6 month and 12 month data will be analyzed using multivariate statistical analyses and groups will be compared on treatment adherence, quit rates and change in weight among abstinent participants. To determine if the association between group assignment and primary outcomes (30-day abstinence and change in weight at 6 months) is moderated by pre-determined baseline and process measures, interaction terms will be included in the regression models and their significance assessed.

**Discussion:**

This study will generate information to inform whether adding weight management to a tobacco cessation intervention delivered by phone, mail and web for smokers seeking help to quit will help or harm quit rates and whether a simultaneous or sequential approach is better at increasing abstinence and reducing weight gain post quit. If proven effective, the combined intervention could be disseminated across the U.S. through quitlines and could encourage additional smokers who have not sought cessation treatment for fear of gaining weight to make quit attempts.

**Trial registration:**

Clinicaltrials.gov NCT01867983. Registered: May 30, 2013

**Electronic supplementary material:**

The online version of this article (doi:10.1186/s12889-016-3231-6) contains supplementary material, which is available to authorized users.

## Background

### Comorbid smoking and obesity are a public health problem

Obesity and smoking account for over three quarters of a million deaths per year in the U.S. [1–3] and about 92 % of smokers have at least one additional health risk behavior [4–8]. More than nine million adults are both obese and smoke [9], markedly increasing their mortality risk [2, 3]. Although effective smoking cessation treatments are available [10–13], weight gain following cessation is common [14–18]. Modest weight gain of about 8-10 lb is the norm, but some smokers gain more than 20 lb after quitting. There is evidence that obese smokers and smokers concerned about post-cessation weight gain may experience greater than average weight gain after quitting and cessation related weight gain can increase the risk for weight related health conditions [16, 19–24]. A systematic review of interventions focused on preventing post cessation weight gain confirmed prior research findings that 80–90 % of smokers gain weight after quitting; that only 10–20 % gain more than 10 kg and that the weight gain is generally permanent unless changes in lifestyle are adopted [25]. The authors concluded that there are very few interventions that entirely prevent post cessation weight gain, but there is evidence of modest effects for reducing the amount of weight gained.

### Efficacy of combined weight and cessation interventions has been established and can help smokers quit while minimizing weight gain

A systematic review of randomized controlled trials that compared combined behavioral smoking and weight management treatment with smoking treatment alone found that the combined interventions produced a significant improvement in cessation and reduced weight gain over tobacco treatment alone in the short term with a similar but non-significant trend at 6 or 12 months [26]. In this review by Spring and colleagues, 779 articles were identified from searching PubMed, Ovid MEDLINE, CINAHL, EMBASE, PsycINFO and Cochrane Central Register of Controlled Trials, and 10 trials met eligibility criteria and were included in the meta-analysis. Of the 2233 adults who participated (2079 females and 154 males), those who received both smoking and weight treatment showed increased abstinence [odds ratio (OR) = 1.29, 95 % confidence interval (CI) = 1.01, 1.64] and reduced weight gain (g = -0.30, 95 % CI = -0.57, -0.02) in the short term (<3 months) compared with patients who received smoking treatment alone. Differences in abstinence (OR = 1.23, 95 % CI = 0.85, 1.79) and weight control (g = -0.17, 95 % CI = -0.42, 0.07) were in the same direction but no longer significant in the long term (>6 months) [26]. One of these studies, also conducted by Dr. Spring and colleagues, showed that a sequential treatment approach which provided cessation treatment first, followed by weight management treatment, reduced weight gain to a greater extent than simultaneous treatment and cessation treatment alone and showed a non-significant trend for better cessation rates [27]. Similarly, Copeland and colleagues tested a sequential approach to weight management following two weeks of cessation treatment and found that individually tailored weight management resulted in higher cessation rates than group treatment. Groups did not differ in weight gain and the study did not have a tobacco cessation only treatment group [28]. According to the most recent Cochrane review, some smoking cessation interventions that included personalized weight management support or very low calorie diets may limit post cessation weight gain without harming abstinence rates, but only in the short term [29]. The review also reported that exercise interventions can reduce weight gain without undermining quit attempts over the long term and that bupropion and fluoxetine were better at suppressing weight gain than NRT but only while using the medications [29].

Most of the successful smoking cessation interventions designed to prevent cessation-related weight gain focused on calorie restriction or meal replacement, behavioral counseling on weight control, some form of exercise and/or cessation medications [29–35]. In addition to dietary and physical activity interventions, a weight acceptance approach has been tested in three trials [32, 36, 37]. The rationale behind this line of research stems from research findings that excessive concerns about weight gain can interfere with successful quitting. Thus the intervention aims to address maladaptive beliefs about weight, encourage acceptance of moderate weight gain and to focus on tobacco cessation. In the original weight concerns trial, Perkins found that adding an intervention aimed at addressing and reducing maladaptive weight concerns simultaneously with smoking cessation treatment resulted in a significant improvement in cessation, as compared to cessation treatment alone. Weight gain among those who quit smoking also differed across groups. At 6 and 12 months, weight gain was significantly lower for the weight concerns group [2.9 ± 2.6 kg and 2.5 ± 4.2 kg] compared with the weight control group [4.1 ± 4.8 kg and 5.4 ± 3.3 kg] and cessation only treatment [6.4 ± 3.5 kg and 7.7 ± 4.7 kg] [36]. The second weight concerns study involved a 2x2 randomized trial offering standard cessation alone vs. the weight concerns intervention crossed with placebo or bupropion. Results showed that among women offered bupropion to help with cessation, those randomized to the weight concerns intervention had significantly greater levels of abstinence at 6 months than did standard smoking cessation counseling combined with bupropion or placebo and a non-significant increase in weight gain at 6 months with no effect on weight at 12 months. A third trial of the weight concerns intervention tested the effectiveness of this weight acceptance approach in a population-based setting of a national tobacco quitline among 2000 male and female smokers seeking help to quit smoking. Results were published after the three systematic reviews and showed that at 6 months the intervention had a significant weight suppressive effect without impacting cessation [37].

These results highlight the difference between “weight concerns” interventions which strive to increase smoking cessation and are not consistently associated with weight suppression after quitting, and “weight loss/weight management” interventions involving diet or targeted behavioral weight loss approaches which do reduce weight gain. Overall, the aggregated findings in these reviews provide no evidence that combining smoking treatment and behavioral weight control undermines either abstinence or weight control and some evidence of benefit for both. Although systematic reviews have established that combining weight management with tobacco cessation treatments are a safe and moderately successful approach for reducing weight gain and improving cessation outcomes in the short term, most trials used intensive, in-person, group interventions and very few included males [25, 26, 29, 38]. Such intensive interventions are unlikely to be adopted at a population level, and if they were, would be unproven for half of the population. A sound knowledge base is needed to inform treatment decisions about best practices for the growing population with multiple health risk behaviors. Since the prior efficacy study conducted by Spring and colleagues suggests that sequential weight control treatment can suppress cessation related weight gain [27], there is a need to determine whether the prior finding is replicable in a population based setting and for both short and long-term tobacco and weight outcomes.

Quitlines provide an ideal setting in which to integrate tobacco cessation with recommended behavioral treatments for other health risk behaviors. Quitlines are freely available in all 50 states and the U.S. territories, have the potential to reach large populations of smokers and have proven to be effective and cost-effective [10–13, 39]. Quitlines offer a range of services including multiple counseling calls with cessation specialists, mailed support materials and access to modest regimens of nicotine patch or nicotine gum (NRT) [13, 39, 40]. Given the risk of both continued smoking and weight gain, it is worthwhile to test successful but intensive combined interventions adapted for population delivery via a telephone quitline. This paper describes the design, methods, adapted interventions and analytic approach for such a clinical trial. The rationale for our study is to replicate and scale the prior efficacy trial described by Spring that tested the simultaneous or sequential addition of weight control treatment compared with tobacco cessation treatment alone [27]. If a combined phone and web based weight control program added to a standard phone/web based cessation treatment had benefits for cessation and weight control, such a program would have substantial reach and public health benefit.

## Methods

### Study design, setting and population

The Best Quit Study is a 3-arm randomized controlled trial in which quitline callers are assigned to one of three treatment groups: standard quitline (cessation treatment alone), quitline plus a weight control treatment added simultaneously, or quitline plus a weight control treatment added sequentially to the cessation program. The study will be conducted at Alere Wellbeing, which operates telephone quitlines as well as a weight management program delivered by phone, mail and web. The tobacco quitline and the weight control program are based on social cognitive theory [41], utilize similar counseling call structures and are supported by comprehensive computer-based tracking and support systems. The combined treatments aim to facilitate use of tobacco and weight control treatments to increase the proportion of smokers who achieve medium (6 months) and long-term (1 year) tobacco abstinence and to minimize weight gain.

The population will include 2550 smokers recruited from Alere Wellbeing’s commercial quitlines (employer groups and health care organizations) and participating state quitlines. Alere Wellbeing operates quitlines for 28 states and more than 800 geographically diverse employer groups and health plans and serves more than 20,000 smokers per month. Study participants will be recruited from individuals who call in to a participating quitline for cessation treatment. Individuals are eligible to participate if they are 18 years or older, smoke 10 or more cigarettes per day (cpd) and are ready to quit smoking in the next 30 days. Exclusion criteria include enrolling in the web-only program, being pregnant or planning to become pregnant within three months, having diabetes, recent or planned weight loss surgery, BMI < 18.5, having an eating disorder, inability to understand English, having no access to the internet or email, being unavailable in the next two weeks, and unwilling to participate in ten counseling calls. As this study takes place in a real-world, phone-based setting, the entire recruitment and enrollment process, including informed consent, will be handled over the phone. Interested individuals who agree to hear more about the study will be transferred to a team of trained research coaches, who will describe the study in detail, obtain informed verbal consent and collect baseline data. Eligible and consenting participants are immediately randomized by a computer generated program into one of three study arms: Standard Care (tobacco only), Simultaneous (tobacco and weight), and Sequential (tobacco followed by weight) and enrolled into the study to begin intervention. A copy of the consent form (See Additional file 1) will be mailed to study participants after enrollment, along with other study materials.

**Fig. 1:**
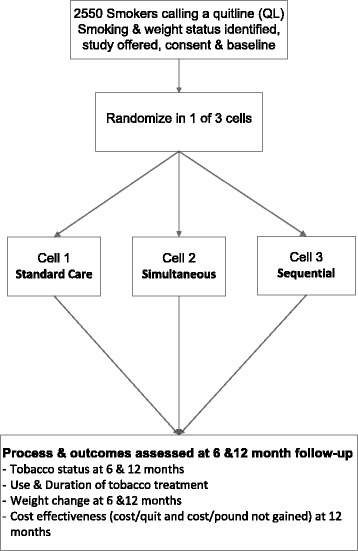
Study design

Process data will be collected using Alere Wellbeing’s automated call tracking system and stored in a secure system. Outcome data will be collected 6 and 12 months after randomization (see Fig. 1: Study Design). We will collect outcome data via surveys administered first by email, then phone, then mailed, for all participants randomized into the study and who do not withdraw consent for follow-up. Strategies to promote follow-up completion include incentives for completing surveys, $2 pre-incentives sent with mailed surveys, and an additional incentive for completing the 12-month email survey within 72 h. Telephone outcome assessors, who will receive specialized training on the study, will be blinded to the intervention assignment. All outcome survey data will be collected via a secure link to DatStat, a survey collection database.

### Interventions

The intervention for all three groups includes 10 coaching calls, mailed materials, and access to one or more web-based programs plus unlimited participant initiated calls for help quitting for up to 12 months from enrollment in the quitline. Standard Care participants receive 5 quit smoking calls followed by 5 calls focused on tips for healthy living (contact control calls). Participants in the simultaneous arm receive 5 calls focused on quitting smoking integrated with the weight management content followed by 5 calls focused on tips for healthy living. Participants in the sequential arm receive 5 quit smoking calls first followed by 5 weight management calls (see Table 1: Call sequence for: Quit Smoking (Q), Weight Management (W), and Contact Control (CC) groups).

**Table 1:** Call sequence for Quit Smoking (Q), Weight Management (W), and Contact Control (CC) calls

Week:	1	2	3	4	5	6	7	8	9	10	11	12	13	14	15
Standard care	Q1		Q2	Q3			Q4			Q5	CC1: Sunscreen	CC2: Preventing the Flu/Hand Washing	CC3: Pedestrian Safety	CC4: Disaster Preparedness	CC5: Energy Savings/Home Efficiency
Sequential	Q1		Q2	Q3			Q4			Q5	W1: Getting Started	W2: Master the Art of Eating Well	W3: Sitting Less, Moving More	W4: Stress and Weight	W5: Wt Call Wrap up
Simultaneous	Q1/W1: getting started		Q2/W2: Master the Art of Eating Well	Q3/W3: Sitting Less, Moving More			Q4/W4: Stress and Weight			Q5/W5: Wt Call Wrap Up	CC1: Sunscreen	CC2: Preventing the Flu/Hand Washing	CC3: Pedestrian Safety	CC4: Disaster Preparedness	CC5: Energy Savings/Home Efficiency

#### The smoking cessation intervention

All participants will receive standard cessation treatment services as dictated by their coverage plan, individual needs, and preferences. This includes the Quit For Life® and state quitline programs, which combines individualized telephone counseling, mailed written materials, and an interactive online program (Web Coach®) designed to complement the phone-based treatment sessions. Participants receive up to 5 counseling calls (1 reactive; 4 proactive), each designed to provide practical expert support to help participants develop problem-solving and coping skills, secure social support, and design a plan for successful cessation and long-term abstinence. As is standard in the quitline programs, participants may be eligible to receive over-the-counter (OTC) and prescription cessation medications, including Nicotine Replacement Therapy (NRT) in the form of patch, gum or lozenge, and varenicline. Because eligibility for cessation medications varies by state or commercial contract, medications may not be provided uniformly across all study participants.

#### The behavioral weight control intervention

The weight management program (Weight Talk®) is a phone and web based weight management-lifestyle program. The program integrates behavioral, psychological and biological science with highly skilled coaching. Content of the intervention comes from physical activity and dietary behavior change interventions proven to be efficacious [42–45]. For this study, Weight Talk® will act as the brief, adapted version of the weight control program used in the prior efficacy study [27]. All coaching calls, the mailed materials and the interactive web encourage and support goal setting and regular self-monitoring of weight, choice of foods, calorie reduction, physical activity, and stress reduction. The recommendations that form the basis of Weight Talk® include the NIH Clinical Guidelines on Identification, Evaluation and Treatment of Overweight and Obesity in Adults as well as the NIH-developed “Dietary Approaches to Stop Hypertension” (DASH) protocol [46]. The DASH diet has been proven to not only help reduce body weight but also to lower blood pressure, blood lipids and diabetes risk factors independent of weight control [46, 47]. The physical activity component of the program is based on the U.S. Public Health Service Guidelines (http://www.ahrq.gov/professionals/clinicians-providers/guidelines-recommendations/tobacco/index.html). The activity component encourages more moving and less sitting with an overall goal of walking 3 to 4 times per week and gradually increasing the time and speed up to > =60 min per session. The stress reduction component helps individuals identify and control stressful situations, find and practice coping skills and monitor progress. The stress reduction module uses evidence based behavioral treatments to help participants reduce the harmful effects of stress that are common in dieters as well as in smokers [48]. The treatment goal is to prevent stress from inhibiting successful behavior change. For this trial, Weight Talk® will be adapted to focus on prevention of weight gain rather than weight loss for people trying to quit smoking. A registered dietician will work with participants to set a calorie goal of approximately 300 cal fewer than their current diet. Reducing calories by this amount is likely to offset the potential weight gain associated with a reduction in metabolism associated with withdrawal of nicotine. Weight will be assessed during each counseling call and calorie adjustments can be tailored to each individual.

#### Healthy lifestyle contact control calls

The standard of care quitline program consists of 5 counseling calls. Because adding Weight Talk® to the smoking cessation treatment in the sequential treatment results in a total of 10 calls, five neutral (contact control) calls need to be added to the standard 5-call quitline group and the simultaneous weight group to equalize the number of contacts. Equality in call number reduces the potential for inequality in subject burden, differential dropout rates and differential response rates to the 6 and 12 month surveys. The ‘contact control’ calls will be delivered by trained coaching staff and the duration is approximately that of a standard care call. The contact control calls will not include weight or tobacco related content. We therefore chose the following ‘neutral’ topics, the content of which is unlikely to affect smoking or a person’s weight:

1. Sunscreen Protection (the coach discusses risks of excessive sun exposure, and behavioral steps a person can take to protect themselves and family).
2. Prevent the Flu/Hand Washing (the coach discusses steps a person can take to reduce their risk of contracting influenza).
3. Pedestrian Safety (the coach provides education about pedestrian safety and tips to increase safety as a pedestrian or driver).
4. Disaster Preparedness (the coach provides a background on the importance of disaster preparedness and provides recommendations on steps a person can take).
5. Energy Savings/Home Efficiency (the coach discusses implications of inefficient energy consumption in homes and steps to reduce costs).

#### Counselor qualifications and training

Separate teams of equally competent counselors will deliver the tobacco or the weight based treatment. Tobacco cessation specialists (quit coaches) will be college educated with at least two years of counseling experience, consistent with prior quitline intervention trials [37, 40, 49]. Weight coaches will also be college educated and have at least two years of counseling experience. Registered dieticians who deliver the second call in the Weight Talk® program will be board certified and experienced. All counselors will have completed at least 100 h of training in the treatment approach as well as ongoing call monitoring and supervision.

### Data safety and monitoring board

The DSMB will consist of at least three independent members who, collectively, have experience in the conduct and monitoring of randomized clinical trials. A quorum will require at least two members. Upon convening, the DSMB will vote on who will serve as the chair. The DSMB membership is restricted to individuals free of apparent significant conflicts of interest. The source of these conflicts may be financial, scientific or regulatory in nature. Thus, study investigators are not members of the DSMB. DSMB members may not own stock greater than value allowed by institutional policy, in or have consulting agreements with a tobacco industry sponsor. The DSMB members will disclose conflicts of interest to fellow members. Any DSMB member who has or develops a significant conflict of interest should resign from the DSMB. DSMB membership is for the duration of the clinical trial. If any members leave the DSMB during the course of the trial, the PI will promptly appoint their replacement. The expected frequency of the DSMB meetings will be twice annually. Ad hoc meetings may be scheduled as needed. The DSMB will hold its first meeting prior to the study launch and will hold its second meeting three months after study launch. The study team will prepare open and closed reports for DSMB members to review on a quarterly basis. The open report will include the study design, protocol amendments, status of dropouts, and compliance. The closed report will be blinded to the PI and will include demographics by study arm, analyses of primary and secondary efficacy endpoints, and severe adverse events (SAEs). The purpose of the DSMB meetings is to review the conduct of the trial to date and assess safety and efficacy of the study intervention. The DSMB will review severe adverse events and determine whether the study should be prematurely discontinued.

### Discontinuing intervention

We will discontinue weight treatment for participants who report during the course of treatment a BMI < 18.5, pregnancy, or other condition that would have made them ineligible for the study at enrollment, however they may continue with the standard tobacco cessation program. There are no restrictions on concomitant care or interventions for the participants during course of the trial.

### Intervention fidelity

To monitor adherence to the cessation and weight intervention protocols (and the healthy living protocols), four raters will assess a 10 % random sample of participants’ audio-taped calls. Treatment fidelity will be assessed using a Call Quality Monitoring Tool (CMT) for the study. Treatment fidelity for Quit Coaches includes evaluation of implementing correct procedures for assessing and dosing of NRT and adherence to treatment protocols according to the PHS Guidelines [10] and recommended theory based topics advised for each call. A similarly rigorous CMT will be used to code recorded calls delivered by the Weight Coaches to assess their adherence to content specific to the weight control intervention, including their ability to utilize Social Cognitive Theory (SCT) to identify and help participants’ overcome their barriers to change [41]. Analysis of treatment fidelity will be performed quarterly. If fidelity for any sampling falls below 80 %, the pool of coaches will receive booster trainings on study content.

### Measures

Data for analyses will come from information collected when a person registers with the quitline, baseline data collected by a quit coach, 6 and 12 month phone or on-line follow-up data collected by a survey team and Alere’s tracking information on number and duration of counseling calls completed.

**Primary outcome measures** will be self-reported 30-day point prevalent abstinence and self-reported change in weight at 6 months.

**Secondary outcome measures** include 7-day point prevalence and continuous abstinence at 6 and 12 months, and reduction in amount smoked in continuing smokers. Other data will include descriptive and process data for analyses of mediators and moderators of intervention effects and cost data.

Survey content will be derived from standardized validated measures used to assess tobacco history, weight variables and outcomes, and from prior research findings and theoretical underpinnings of efficacious clinical trials [27, 33, 34, 37, 40, 50]. Most of the following measures will be assessed at baseline and/or 6 & 12 months and used to describe participants and evaluate outcomes.

*Demographics:* age, gender, race, ethnicity, education, height, weight, and other general demographics.

*Chronic Disease:* assessed by asking participants if they have ever been diagnosed with diabetes, heart disease, asthma, or a lung disease.

*Depressive symptoms:* measured with the PHQ-2, a 2 item screening measure for depressive symptoms that has demonstrated strong criterion and construct validity. The scale uses a 4-point response option to assess frequency of dysphoria and anhedonia. Sensitivity of the PHQ-2 has been reported at 84 %, with 72 % specificity for a cut score of 3 [51].

*Perceived stress:* measured with the 4-item Perceived Stress Scale (PSS), to assess the degree to which respondents find their lives to be stressful. The PSS has been shown to be a valid and reliable measure with alpha reliability coefficients from .84 to .86 [52]. PSS scores have been shown to be related to smoking relapse [53].

*Smoking/quit attempt history:* lifetime and current smoking, cigarettes smoked per day, history of quit attempts, prior use of nicotine replacement and other cessation treatments, and exposure to other household users.

*Nicotine dependence:* Fagerström Test of Nicotine Dependence (FTND) [54], which is the most commonly used measure for assessing nicotine dependence and is predictive of abstinence [55].

*Self-efficacy for quitting:* using the question “How confident are you that you could quit smoking for good?” which has been used to assess confidence and predict smoking cessation [56].

*Self-efficacy for preventing weight gain:* using the weight concerns questionnaire [57].

*Weight concerns:* assessed with 2 questions: “On a scale of 0–100, how concerned are you about gaining weight after quitting?” and “On a scale of 0–100, how concerned would you be if quitting smoking caused you to permanently gain 10 lb?”

*Physical and sedentary activities –* using standard measures to assess days per week (and minutes/day) in moderate/vigorous activity.

*Dietary behaviors:* assessed by asking about the number of fruits and vegetables consumed in a typical day and the number of days per week participants include at least 5 fruits and vegetables.

*Treatment utilization:* assessed via automated records of use of Alere Wellbeing counseling calls, the website, pharmacotherapy, and other services provided by the tobacco quitline. Utilization of treatments not provided through the study will also be assessed in the follow-up survey.

*Smoking outcomes:* assessed at 6 and 12 months to characterize 7- and 30-day point prevalence reports of nonsmoking and continuous abstinence using definitions recently recommended by the Society for Research on Nicotine and Tobacco (SRNT) [58].

*Reduction in amount smoked:* asked for those who continue to smoke at 6 months or 12 months and calculated as the difference in cigarettes smoked per day at baseline and follow-up.

*Post-cessation weight gain:* the difference between self-reported weight at registration/baseline and follow-up.

*Outcome expectations for success at smoking cessation and weight control:* single-item 10-point scales to rate participants’ expectations about the degree to which treatment would (a) help them quit smoking, (b) minimize weight gain, and (c) be effective overall. In the original efficacy trial [27], one-month test–retest reliabilities for these single-item scales ranged as follows: .36–.71 for outcome expectations about quitting smoking, .42–.59 for expectations about minimizing weight gain, and .34–.74 for expectations about the program overall, with ratings showing increased reliability later in treatment. Data from the original efficacy trial indicates that adding weight management increased outcome expectations for abstinence [27]. We will assess whether groups are similar at baseline on these measures and will evaluate these measures in mediator analyses.

### Sample size and power analysis

Alere’s historical 30+ day self-reported abstinence rate at 6 months based on an intent to treat protocol (all eligible for survey are included) is 29.6 % for employer and health plan clients and 20 % for state quitlines. The lower quit rates for state quitlines likely reflects socioeconomic differences (eg more uninsured smokers and lower education and income). Survey response rates are also lower in state quitlines. By enrolling 850 participants per treatment group, we expect to have 80 % power to detect a difference in the abstinence rate between the standard quitline control condition and the combined treatment groups of 5.7 % or greater (ie an odds ratio of 1.30) using a Fisher’s exact test. Similarly, we can detect a difference between standard care and either of the combined treatment groups of 6.5 % (OR = 1.34) or greater. The detectable odds ratio may be slightly smaller when testing for treatment effectiveness using logistic regressions controlling for baseline BMI, gender, age, and nicotine dependence. We estimate that at 12 months the abstinence rates will decrease by 3 % in each group, resulting in 80 % power to detect a change of 5.5 % (ie an odds ratio of 1.31). Effect sizes from other studies have shown that an increase in quit rates of 2.5 % or greater are clinically significant. For example, the quit rate without advice from a physician is 7.9 % versus 10.2 % with physician advice [10]. Our detectable effect size is also comparable to the effects of many different treatments that are widely applied in clinical practice. For example, without help, individuals have an estimated 10.8 % abstinence rate versus 13.1 % with proactive telephone counseling, 13.9 % with group counseling, and 16.8 % with individual counseling [10]. Since behavioral counseling is recommended, this demonstrates that increases on the order of 2.3 to 6.0 % are clinically relevant. Data from Alere’s standard services experience and a prior study were used to calculate the standard deviation of calculated post-treatment abstinence rates [37].

### Statistical analysis plan

*Aim 1.* The primary cessation and weight change outcomes will be assessed at 6 months and the secondary outcomes at 12 months. We hypothesize that combined treatments will be more effective than standard treatment and that sequential treatment will be more effective than simultaneous treatment for increasing quit rates or reducing weight gain, without harming the other outcome. The primary analysis approach for testing the effectiveness of the combined treatments on cessation will be logistic regression with an indicator for the main effect of treatment and an interaction term to account for the difference in treatment effectiveness of the two treatments. The regression equation will also include baseline characteristics that are predictive of abstinence (age, gender, etc.) and an indicator of whether the participant is qualified for receiving NRT through their State quitline plan. Analyses of cessation will be conducted three ways: 1) using multiply imputed values for missing cessation status; 2) on the set of responders to follow-up (ie, those for whom we know the cessation outcome), 3) assuming that non-responders (lost to follow-up) have relapsed. We will also conduct sensitivity analyses for missing cessation results by reanalyzing the data with different assumed probabilities of relapse. The primary analysis approach for testing the effectiveness of the combined treatments on weight will be linear regression. The regressions will include an indicator for the main effect of treatment, an interaction term to account for differences in treatment effectiveness, current cessation status and the interaction of treatment group and current cessation status as covariates, thus allowing estimates of weight gain for all participants and (as secondary analyses) for quitters and smokers. A regression approach that is robust to heterogeneity in variance will be used. We will address missing values using multiple imputation as well as conducting sensitivity analyses with different assumed amounts of weight gain for non-responders to determine how extreme such weight gains (or differential weight gains among non-responders in different groups) would need to be to invalidate statistically significant results.

For all of the aims, preliminary analyses will include summary statistics (mean, standard deviation, skewness, kurtosis, range), cross tabulations of baseline variables and outcomes for each group, correlations among variables, and plots of outcome variables versus time. Standard regression diagnostics will be performed including examination for heteroscedasticity, outliers, observations with large leverage, observations with large influence, and normality of residuals (where appropriate). Outcome variables may be transformed to improve the normality of residuals. Plots (such as residuals versus covariates and residuals versus fitted values) will be generated to examine the model fit. The Benjamini-Hochberg procedure will be used to adjust the p-value for multiple comparisons separately for primary and secondary measures. The general analysis approach for the secondary measures will be repeated measures logistic, ordinal, Poisson, or linear regression depending on the type of outcome measure [59].

*Aim 2* involves analyses to test the cost-effectiveness of the combined interventions over standard care for cessation and for weight. We hypothesize that combined treatments are more cost effective than standard treatment and sequential more cost-effective than simultaneous for cessation and weight. With respect to cost per quitter, cost-effectiveness analyses will be conducted to quantify: 1) the cost per quitter for the standard and combined treatment, 2) the incremental cost per quitter of the combined treatment (relative to the standard cessation program), and 3) the relative cost-effectiveness of the combined treatment versus other treatments in the literature. Treatment costs will be calculated on the basis of Alere’s employer and state reimbursements for the smoking cessation program (which may include NRT treatment) and weight management treatment. In addition, we will calculate the cost per quality adjusted life year gained using the approach described by Javitz [60]. With respect to weight gain, cost-effectiveness analysis will be conducted to calculate the cost per pound not gained (ie, the additional cost of the weight management treatment divided by the difference between the weight gain in the standard and combined treatment group) and this cost will be compared to medical costs associated with weight gain from the literature [61–63] and to the cost per pound lost from commercially available weight reduction programs (since presumably individuals in the standard quitline group could use such programs to lose weight or prevent weight gain).

*Aim 3* includes analyses to evaluate theoretically pre-specified mediators of treatment effects on cessation.

To test whether changes attributable to the weight management program mediate the cessation rate, we will examine changes in weight concerns, weight, outcome expectancies on quitting, and self-efficacy in quitting without weight gain. Mediation implies a causal hypothesis whereby an independent variable (ie, the weight management program) causes a mediator (eg, reduced weight concerns) that causes a change in a dependent variable (ie, cessation) [64]. We will follow four steps to test mediation effects [65, 66]. For example, to assess whether reduced weight concerns mediates cessation, we must: (1) demonstrate significantly different cessation outcomes for weight management and comparison groups, (2) demonstrate differences in weight concerns by condition, (3) establish that reduced weight concerns is significantly related to cessation rate, and (4) show that the effect of a weight management intervention on cessation rate is significantly less after controlling for change in weight concerns. We will further test whether changes in outcomes expectancies of treatment on quitting, self-efficacy in quitting without weight gain, physical activity and diet (eg fruits and vegetables) mediate the effect of the weight management program on weight gain. Regression analyses will be conducted to examine the moderating effects on cessation and weight gain of baseline characteristics of BMI, age, gender, weight concerns, nicotine dependence, outcome expectancies and self-efficacy in quitting. Moderators will be included in the regression analyses as mean-centered covariates and as interaction terms with the combined treatment group indicator.

Exploratory analyses will also assess mediators of weight gain and whether baseline BMI, weight concerns, self-efficacy, outcome expectancies, nicotine dependence, use of medications, age and gender moderate the effectiveness of the different interventions for cessation and weight outcomes.

All analyses will be conducted using SAS or STATA software.

## Discussion

This paper describes the Best Quit Study which compares tobacco cessation treatment alone or combined tobacco and weight control treatment for improving the dual outcomes of abstinence and weight gain prevention and tests whether a simultaneous or sequential approach is more effective and cost effective than standard tobacco cessation treatment alone. The trial attempts to replicate a prior efficacy trial by translating and adapting the interventions for delivery within a population based setting of a quitline. Potential limitations include differences between the implemented effectiveness trial and the original efficacy trial and the use of self-reported outcomes. We have translated the original, in-person, group based intervention to accommodate the real-world phone based treatments offered through the quitline. We did not offer meal replacement food, but our Weight Talk® intervention includes a mailed education packet with an activity tracker and charts to monitor weight and physical activity. As such, the resultant intervention has the potential for broad reach and widespread adoption. The use of self-reported, non-validated outcome measures, remain a potential limitation. Using blinded non-study survey professionals for delivering the phone based follow-up surveys have been shown to reduce bias in reporting smoking status and weight [58, 67, 68].

## Conclusions

The trial was successfully implemented and was acceptable to quitline participants. Our experience replicating the original efficacy trial and adapting the interventions for delivery through existing national quitlines was presented at the European Society on Nicotine and Tobacco Research in Santiago De Compestella, Spain in September 2014 [69]. The study results will be reported according the CONSORT statement for cluster randomized trials [70].

### Public health impact

Given the national epidemic of obesity and the costs of smoking among overweight adults, population-based smoking cessation treatments are needed that increase abstinence and prevent weight gain. This study could identify a strategy for translating efficacious smoking and weight control interventions from research to practice (ie through standard quitline operations), thereby increasing the reach and impact of combined treatment in the population. Results will address the question of whether a simultaneous or sequential intervention on multiple behaviors (smoking cessation, diet and exercise) is helpful or harmful to cessation or weight outcomes. If either a simultaneous or sequential combined intervention improves cessation and limits weight gain, dissemination of such interventions could have a significant scientific, clinical and public health impact by encouraging more quit attempts in the population, reducing relapse, improving long-term health outcomes, and reducing weight-related comorbidities, long-term medical costs, and costs associated with post-study weight reduction programs. This study will be an important replication test of prior efficacy studies in that it uses a more acceptable setting (quitlines) that contains existing tobacco and weight control services.

## Abbreviations

BMI, body mass index; CC, Contact Control (Calls); CI, confidence interval; CMT, call monitoring tool; cpd, cigarettes per day; DASH, Dietary Approaches to Stop Hypertension; FTND, Fagerström Test of Nicotine Dependence; g, gram; Kg, kilogram; NIH, National Institutes of Health; NRT, nicotine replacement therapy; OR, odds ratio; OTC, over-the-counter; PHQ-2, Patient Health Questionnaire; PHS, Public Health Service; PSS, Perceived Stress Scale; Q, Quit Smoking (Calls); SCT, Social Cognitive Theory; SRNT, Society for Research on Nicotine and Tobacco; U.S., United States; W, Weight Management (Calls).

## Additional file

Additional file 1: BQS Consent–mailed version. (DOCX 30 kb)
