# Supplementary material for: Comparative effectiveness of adding weight control simultaneously or sequentially to smoking cessation quitlines: study protocol of a randomized controlled trial
Source: BMC Public Health. 2016 Jul 22;16:615. doi: 10.1186/s12889-016-3231-6 (PMC4957297; doi:10.1186/s12889-016-3231-6)
Supplement: Additional file 1: — BQS Consent–mailed version. (DOCX 30 kb) [file 12889_2016_3231_MOESM1_ESM.docx]

**BQS CONSENT – MAILED VERSION**

September 23, 2013

Dear [PARTICIPANT NAME],

Thank you for your interest in the Best Quit Study! By law, we are required to provide you a copy of this information so you know your rights as a study participant.

**PURPOSE**

- Some people gain weight when they quit smoking and this can be a barrier to quitting. We don’t know if trying to quit and working on your weight at the same time will be as beneficial as just focusing on quitting.
- The purpose of the Best Quit Study is to compare three different ways that we can help people quit smoking and manage their weight.
- For this study, in addition to the Quit For Life program offering 5 calls to help you quit smoking, you will receive an extra 5 calls of varying content.
- Some people in the study will receive extra calls to discuss weight, some will receive extra calls to discuss general health education topics, and some will receive a combination. You will have an equal chance of being randomized into one of three groups.

**Group descriptions**

- All three groups will receive a total of 10 coaching calls, mailed materials, and access to one or more web-based programs.
  - One group receives 5 quit smoking calls followed by 5 calls focused on tips for healthy living. This group receives information about weight management after completion of the study.
  - One group receives 5 quit smoking calls followed by 5 weight management calls. And,
  - One group receives 5 calls focused on quitting smoking and weight management followed by 5 calls focused on tips for healthy living.
- You will be contacted at 6 and 12 months to complete follow-up surveys. Each survey takes about 10 minutes. We will send you a $15 gift card each time you complete a survey.
- Only one person per household can participate in this study.
- Your participation is voluntary and you may refuse to participate or dropout of the study at any time. Whether or not you choose to participate in the study you are still eligible to receive services from the Quit For Life program.
- By participating in this study, you have informed the research staff that in the next six months, you are not planning to be away from your phone or unavailable to take calls for longer than 3 weeks.

**Potential study risks**

- While you are quitting smoking you may experience some symptoms of nicotine withdrawal such as irritability.
- If you are assigned to a group receiving weight management calls you may experience some discomfort due to changes in your diet or physical activity.
- You may experience some level of emotional discomfort during discussion of weight.
- There is a small risk that some personal information could be disclosed to members of our research team at Northwestern University or SRI International. We have strict methods to protect your information, which we will discuss in a moment. We may share recordings of your calls with members of the study team for quality assurance purposes.
- We will be sending you a few study related emails to the email address you provided a few minutes ago. As with all email, there is some risk of unauthorized access.

**study benefits**

- You will receive additional counseling calls which may increase your success in quitting smoking.
- You may develop skills that will help you live a healthier lifestyle.
- You may experience less weight gain after quitting which can have health benefits.
- You may feel a benefit from participating in a study which could help other people quit smoking in the future.

**PRIVACY PROTECTION**

- Any personal information you provide to us will be kept in locked cabinets and computer workstations. Both will be secured from unauthorized access. Your name and personal information will never be published.
- If you have questions about this study you can contact Dr. Terry Bush at 206-876-2201. Or write to: Alere Wellbeing, Suite 2100, 999 3rd Avenue, Seattle, Washington 98104.
- If you have questions about your rights as a study participant you can contact the Western Institutional Review Board (WIRB) which is a group of people who perform independent review of research. Their phone number is: 1-800-562-4789.
